# Interstudy reproducibility of the second generation, Fourier domain optical coherence tomography in patients with coronary artery disease and comparison with intravascular ultrasound: a study applying automated contour detection

**DOI:** 10.1007/s10554-012-0067-8

**Published:** 2012-05-26

**Authors:** Z. Jamil, G. Tearney, N. Bruining, K. Sihan, G. van Soest, J. Ligthart, R. van Domburg, B. Bouma, E. Regar

**Affiliations:** 1 Thoraxcenter, Bd 585, Erasmus MC, Dr. Molewaterplein 40, 3015-GD Rotterdam, The Netherlands; 2 Wellman Center for Photomedicine, Boston, MA USA; 3 Department of Pathology, Massachusetts General Hospital, Boston, MA USA; 4 Department of Dermatology, Massachusetts General Hospital, Boston, MA USA; 5 Harvard-MIT Health Sciences and Technology, Cambridge, MA USA

**Keywords:** Interstudy variability, Fourier domain, Optical coherence tomography, Intravascular, Ultrasound, Reproducibility

## Abstract

Recently, Fourier domain OCT (FD-OCT) has been introduced for clinical use. This approach allows in vivo, high resolution (15 micron) imaging with very fast data acquisition, however, it requires brief flushing of the lumen during imaging. The reproducibility of such fast data acquisition under intracoronary flush application is poorly understood. To assess the inter-study variability of FD-OCT and to compare lumen morphometry to the established invasive imaging method, IVUS. 18 consecutive patients with coronary artery disease scheduled for PCI were included. In each target vessel a FD-OCT pullback (MGH system, light source 1,310 nm, 105 fps, pullback speed 20 mm/s) was acquired during brief (3 s) injection of X-ray contrast (flow 3 ml/s) through the guiding catheter. A second pullback was repeated under the same conditions after re-introduction of the FD OCT catheter into the coronary artery. IVUS and OCT imaging was performed in random order. FD-OCT and IVUS pullback data were analyzed using a recently developed software employing semi automated lumen contour and stent strut detection algorithms. Corresponding ROI were matched based on anatomical landmarks such as side branches and/or stent edges. Inter-study variability is presented as the absolute difference between the two pullbacks. FD-OCT showed remarkably good reproducibility. Inter-study variability in native vessels (cohort A) was very low for mean and minimal luminal area (0.10 ± 0.38, 0.19 ± 0.57 mm^2^, respectively). Likewise inter-study variability was very low in stented coronary segments (cohort B) for mean lumen, mean stent, minimal luminal and minimal stent area (0.06 ± 0.08, 0.07 ± 0.10, 0.04 ± 0.09, 0.04 ± 0.10 mm^2^, respectively). Comparison to IVUS morphometry revealed no significant differences. The differences between both imaging methods, OCT and IVUS, were very low for mean lumen, mean stent, minimal luminal and minimal stent area (0.10 ± 0.45, 0.10 ± 0.36, 0.26 ± 0.54, 0.05 ± 0.47 mm^2^, respectively). FD-OCT shows excellent reproducibility and very low inter-study variability in both, native and stented coronary segments. No significant differences in quantitative lumen morphometry were observed between FD-OCT and IVUS. Evaluating these results suggest that FD-OCT is a reliable imaging tool to apply in longitudinal coronary artery disease studies.

## Introduction

Optical coherence tomography (OCT) is a relatively new, but rapidly accepted invasive coronary imaging tool [1]. As it requires good spatial coherence of the near infrared light beam to create high resolution [2], cross sectional images of the coronary artery, it requires transient clearing of the coronary during image acquisition [3]. This prerequisite hampered widespread use of the first generation time-domain OCT (TD-OCT) systems in the past, which required proximal balloon occlusion and simultaneous distal flush delivery during pull-back of the OCT imaging probe [4]. Recently, a second generation of the technology, Fourier domain OCT (FD-OCT), has been introduced for clinical application to overcome these limitations. FD-OCT allows high speed data acquisition, both in terms of frame rate (>100 frames/s) and in pullback speed (between 20 and 40 mm/s) and alleviates the need for transient balloon occlusion. In consequence, a long coronary artery segment can be rapidly imaged within few seconds and without introducing ischemia during imaging [5]. The older TD-OCT method was only able to acquire images at a maximum of 30 frames/s and a used pullback speeds up to 3 mm/s, requiring a pullback time of 10 s per 30 mm of coronary artery. The introduction of FD-OCT has sparked widespread application in clinical practice and research. Its high resolution, excellent image quality and the high contrast between lumen and vessel wall have proven to provide highly accurate [6] morphometry in vivo, both in terms of accuracy when compared to histomorphometry as well as in terms of inter- and intra-observer variability [7–11].

Until today, intravascular ultrasound (IVUS) is still the reference method in longitudinal intravascular imaging driven studies. In contrast to OCT it uses an acoustic wave to create cross-sectional images of the coronary artery. To apply FD-OCT in such longitudinal studies it is important to know its inter-study variability of quantitative measurements. The reproducibility of fast data acquisition, as required in the FD-OCT imaging protocol, with intracoronary flush application is poorly understood. Therefore, we investigated the inter-study variability of FD-OCT in both native and stented coronary segments, and compared OCT morphometry to intravascular ultrasound (IVUS).

## Methods and materials

### Study population

We included 18 patients with angina and/or objective evidence of ischemia, who were scheduled for percutaneous coronary intervention. The study protocol was approved by the local medical ethics committee. Patients with acute myocardial infarction, hemodynamic instability, renal insufficiency, allergy to X-ray contrast, left main stem or ostial right coronary artery lesions, bifurcation lesions, venous bypass graft lesions, chronic total occlusions, last remaining vessel or extremely tortuous vessels were excluded.

**Fig. 1:**
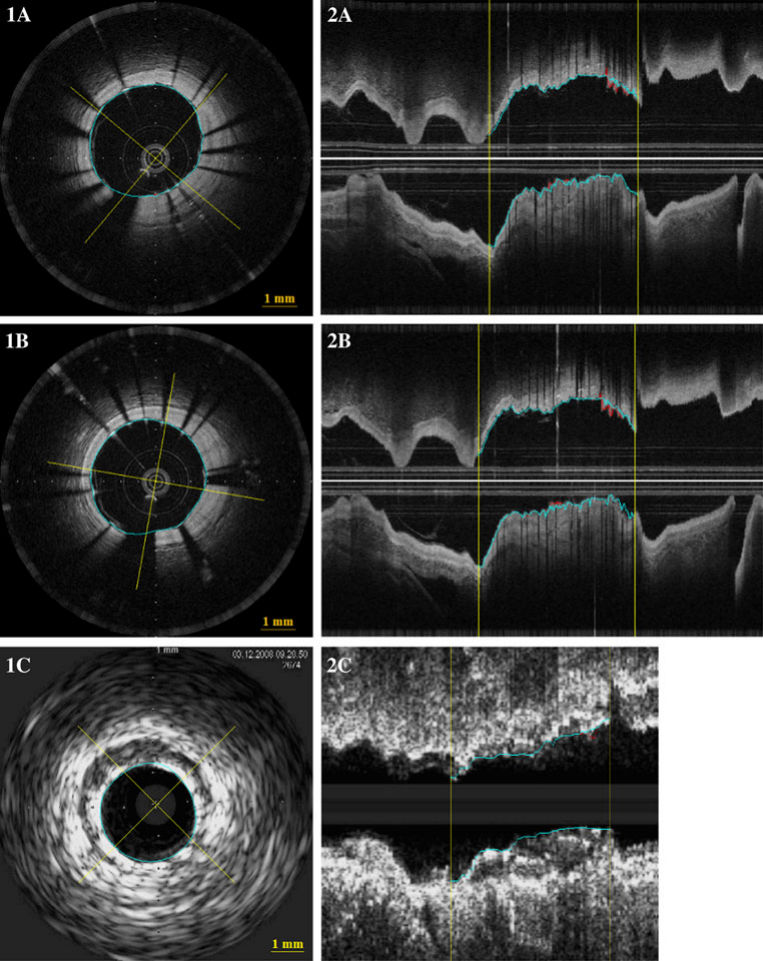
Corresponding cross sectional (**1**) longitudinal (**2**) FD-OCT (**A**, **B**) and IVUS (**C**) images. The *yellow lines* on 2 indicate the region of interest. The *blue lines* indicate stent area and the red lines indicate lumen area

Patients underwent the following procedures in the catheterization laboratory: Coronary angiography, treatment of culprit lesion (PCI according to local standards), and invasive imaging in random order: FD-OCT imaging (test series), FD-OCT imaging (retest series), IVUS imaging. We performed 10 FD-OCT pullbacks in native coronaries (n = 5 test, n = 5 retest) (Cohort A) and 26 FD-OCT pullbacks in stented coronary segments (n = 13 test, n = 13 retest) (Cohort B). For the comparison with IVUS we used 10 FD-OCT pullbacks and 10 matched IVUS pullbacks. The corresponding regions of interest were selected for morphometry using side branches and stent edges as landmarks (Fig. 1).

### FD-OCT imaging system

OCT imaging was performed with a non-commercial, FD-OCT system, as described in detail elsewhere [12, 13]. This system used a wavelength-swept laser with a center frequency of 1,310 nm as a light source. The FD-OCT imaging catheter had a short monorail design with a catheter profile of 2.4Fr compatible with 6F guiding catheters The FD-OCT imaging catheter contained a fiber-optic imaging core covered by and withdrawn within a translucent sheath at a pullback speed of 20 mm/s. Data were processed in real-time and stored digitally.

#### FD-OCT data acquisition

We used standard femoral approach in all patients. Weight adapted, unfractionated heparin was given to maintain an activated clotting time (ACT) >300 s. After placement of the guiding catheter (6F) into the coronary ostium, a standard PCI guide wire was advanced into the coronary artery in conventional manner. After administration of nitrates (0.2 mg NTG ic), the FD-OCT imaging catheter was advanced into the coronary artery. Radiopaque markers at the distal catheter tip and at the imaging core allowed positioning of the optical probe distal to the region of interest. After FD-OCT catheter placement, blood was cleared by injection of iso-osmolar contrast (Iodixanol 370, Visipaque™, GE Health Care, Ireland) at 37 °C with an injection pump (Mark-V ProVis, Medrad, Inc. Indianola, PA, USA; flow rate 3 ml/s) through the guiding catheter. The FD-OCT pullback was started as soon as the artery was cleared from blood and stopped when the imaging core reached the guiding-catheter. After successful completion of the first FD-OCT pullback, the imaging catheter was withdrawn within the guiding catheter. Then the FD-OCT imaging catheter was re-advanced into the coronary artery for the second pullback as described above.

### FD-OCT analysis

To evaluate the inter-study variability the two FD-OCT pullbacks were analyzed independently. The OCT pullbacks were first converted to the standard medical imaging format, e.g. DICOM, using a custom viewing and conversion package called OFDEye. Then the lumen contour was drawn using ‘automatic contour detection’ available in MATLAB software [14]. The stent contour was traced using a multiple point detection option in MATLAB software. Corrections to the lumen and stent contour were made where necessary and then the contour data was exported to CURAD (vessel analysis, CURAD BV, Wijk bij Duurstede, The Netherlands) [15] for further analysis. Side-branch containing cross-sections were not excluded. For each cross-section, the enclosed area of the lumen contour was calculated. Frames showing a relatively large deviation in areas compared to their neighbors were labeled as incorrect. A search and substitute algorithm replaced these contours by the closest available correct contour in the longitudinal direction. And these were corrected manually where needed.

#### Z-offset correction

**Fig. 2:**
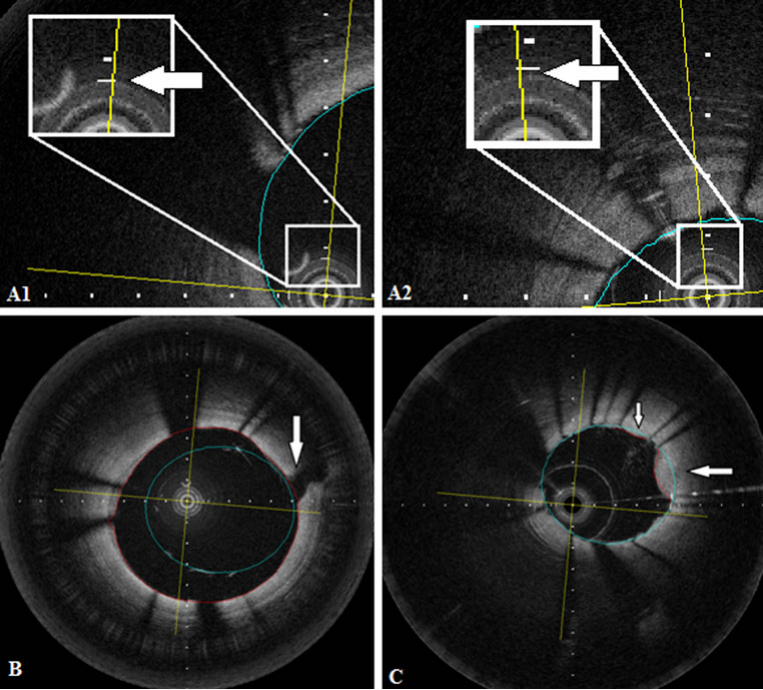
**A1** and **A2** show cross sectional images of two different pullbacks where the z-offset is slightly different. A1 has a correct z-offset and **A2** shows a image of a pullback where the z-offset needs to be corrected. **B** Strut malapposition was defined as presence of at least one strut separated from the vessel wall (not being a side branch), if the distance between the endoluminal reflection of the strut and the vessel wall was larger than the thickness of the stent strut and polymer. The *arrow* in **B** shows a side branch. The *blue line* indicate stent area and the *red line* indicate lumen area. **C** Example of tissue protrusion: defined as convex shaped tissue between the stent struts without disruption of the continuity of the endoluminal vessel surface strut. The *arrow* in **C** shows two areas of tissue protrusion

During image acquisition, the optical fiber in the catheter core can stretch. This may produce changes in the size of the z-offset along the pullback that can affect the accuracy of the measurements (Fig. 2A). Therefore, the z-offset was checked and modified if necessary in all the pullbacks before performing any quantitative measurement. After image acquisition the z-offset was checked again using the OFDEye viewer and corrected for the complete pullback in a frame in which the FD-OCT imaging catheter sheath was in direct contact with the vessel wall, based on aligning the FD-OCT imaging catheter sheath and the vessel wall with the fiducials.

### IVUS imaging

IVUS imaging was performed after administration of nitrates (0.2 mg NTG ic) using commercially available mechanical (Atlantis, Boston Scientific, MA, USA) or phased array transducer systems (Volcano eagle eye, Volcano Corp, Rancho Cordova) as described elsewhere in conventional manner, using an automated pullback device operating at 0.5 mm/s [16].

### IVUS analysis

All IVUS pullbacks were first gated by the validated Intelligate method [17], which automatically selects the end-diastolic frames only to form a new pullback. The gated pullbacks have a smooth appearance of the coronary instead of the rough appearance in non-gated IVUS, which helps to improve accuracy. The lumen and stent area were analyzed using previously validated dedicated quantitative IVUS analysis software (Vessel analysis, CURAD B.V., Wijk bij Duurstede, Netherlands) [15].

### Invasive imaging pullback analysis

Both, for repeated FD-OCT pullbacks, as well as for the corresponding IVUS pullbacks, the same regions of interest were selected for morphometry using vessel analysis software available in CURAD, using side branches and stent edges as landmarks (Fig. 1).

### Definitions

The definitions of the lumen measurements, stent measurements, strut malapposition (Fig. 2B), tissue protrusion (Fig. 2C) and relocation are found in Table 1.

**Table 1:** Definitions

Lumen area (mm^2^)	The surface of the lumen clearly visualized during flush administration as dark, signal poor region delineated by the most inner, signal intense endoluminal leading edge
Mean lumen area (mm^2^)	The mean of lumen areas of all frames in the selected ROI
Minimal lumen area (mm^2^) (MLA)	The smallest lumen area in the selected ROI
Relocation of minimal luminal area (MLA)	In those cases which the longitudinal position of MLA of first, test pullback or OCT was located in a different position in the second, retest pullback or IVUS. For the comparison of the position of the MLA between the two repeated pullbacks, the distance of the frame with the MLA from the distal starting point of the ROI was noted in the test series. Then, the position of the frame with the MLA was expressed as length percentage of the total ROI. The same procedure was performed in the re-test series and IVUS pullbacks
Stent struts	(1) Highly reflective surfaces (metal) that cast dorsal, radial shadows (2) Highly reflective surfaces without dorsal shadowing (3) Sector shaped shadows with sharp defined borders radial to the lumen
Start and end of stent	The first and the last frame with circumferentially visible struts
Stent area (mm^2^)	The surface of the stent by tracing individual stent struts assuming a circular shaped lumen
Mean stent area (mm^2^)	The mean of stent areas of all frames in the selected ROI
Minimal stent area (mm^2^) (MSA)	The smallest stent area in the selected ROI
Relocation of minimal stent area (MSA)	Was assessed as described for relocation of minimal lumen area
Tissue protrusion	Convex shaped tissue between the stent struts without disruption of the continuity of the endoluminal vessel surface strut
Strut malapposition	Presence of at least one strut separated from the vessel wall (not being a side branch), if the distance between the endoluminal reflection of the strut and the vessel wall was larger than the thickness of the stent strut and polymer [31, 34]

#### Relocation

Relocation of the MLA or MSA was considered significant if the difference between the two OCT pullbacks (test and retest) or the OCT and IVUS pullback was >10 % and if the MLA or MSA had a >10 % difference in the normalized longitudinal position.

### Statistical analysis

Statistical analysis was performed using SPSS 17.0 for Windows (SPSS, Chicago, IL, USA) and MedCalc 11.5. Continuous variables are expressed as mean ± 95 % CI or median and interquartile range if appropriate. Categorical variables are expressed as percentages. The absolute and relative differences between measurements obtained with the different techniques were calculated. The relative difference was defined as the absolute difference between repeated pullbacks divided by their average. Data are also expressed in Bland–Altman plots [18] showing the difference between corresponding lumen measurements for both techniques.

The OCT data were presented as per-frame analysis and as per segment or as per stent analysis. In the per frame analysis the morphometry of corresponding frames was compared individually. The per-frame analysis reflects the variability between repeated measurements at corresponding locations within the coronary artery.

The per-segment analysis compares the mean lumen and stent areas as well as their minimal areas and reflects clinically relevant measures for the smallest lumen and stent expansion within a pullback.

## Results

Invasive imaging was successfully performed in 20 coronary arteries (LAD n = 14, RCA n = 3, LCx n = 3). No imaging related complications were observed.

Quantitative OCT analysis using an automated contour detection software was successful in all arteries. Manual adjustment of the automated contour tracing was required in 15 % of the cross-sections in our series. Manual adjustment was primarily necessary cross sections with multiple or large side-branches or in the stented segments when only few stent struts were visible in a particular cross-section, e.g. due to non centered, non-co axial OCT catheter position in bended or tortuous coronary segments.

### Cohort A: FD-OCT native coronary segments

**Fig. 3:**
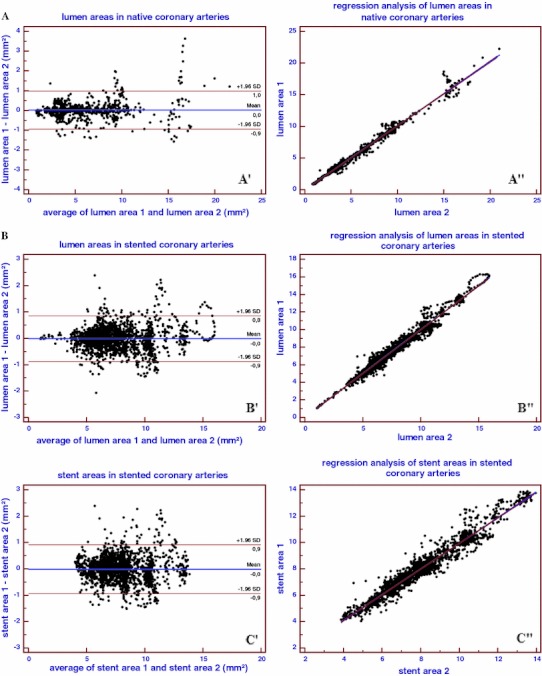
**A** Per frame (n = 1,472) analyses in native coronary arteries. Bland–Altman plot (**A′**) showing the differences in lumen areas between two corresponding pullbacks. Regression analyses line (**A″**) showing correlation between corresponding lumen areas per frame. **B** Per frame (n = 3,520) analyses in stented coronary arteries. Bland–Altman plots showing the differences in lumen areas (**B′**) and stent areas (**C′**) between two corresponding pullbacks in stented coronary arteries. Regression analyses lines showing correlation between corresponding lumen areas (**B″**) and stent areas (**C″**) per frame

A total of 1,472 frames were included into the analysis. 736 frames from the test series (n = 5 pullbacks), and 736 matching frames from the retest series (n = 5 pullbacks). Per frame analysis revealed a very low inter-study variability for mean lumen area of 0.02 ± 0.04 mm^2^ (0.37 %). Figure 3A shows the Bland–Altman plot and linear regression analysis.

**Fig. 4:**
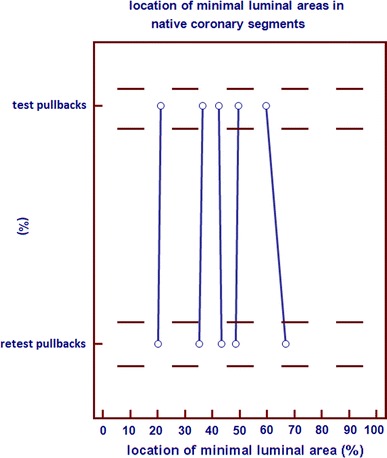
Comparison of the longitudinal position of minimal luminal area (MLA) in native coronary segments between two corresponding OCT pullbacks. X-axis: length percentage of the total ROI/longitudinal position. Y-axis: corresponding pullbacks

Likewise, per coronary segment analysis (mean test pullback length = 182 frames; mean retest pullback length = 177 frames) demonstrated an inter-study variability for mean lumen area of 0.10 ± 0.38 mm^2^ and for minimal luminal area of 0.19 ± 0.57 mm^2^ (Table 2). There was no case of relocation of the MLA (Fig. 4).

**Table 2:** Inter-study variability in native coronary segments (per segment analysis)

	Mean lumen area	Minimal luminal area
OCT pb1	6.74 ± (3.99) mm^2^	3.24 ± (3.85) mm^2^
OCT pb2	6.64 ± (3.73) mm^2^	3.05 ± (3.30) mm^2^
Pullback 1 versus pullback 2		
Absolute difference	0.10 ± (0.38) mm^2^	0.19 ± (0.57) mm^2^
Relative difference	1.55 %	6.11 %
Linear regression		
Slope	1.07	1.16
Intercept	−0.35	−0.31
R^2^	0.99	0.99
*P*	<0.001	<0.001

### Cohort B: FD-OCT stented coronary segments

A total of 3,520 frames were included into the analysis. 1,760 frames from the test series (n = 13 pullbacks), and 1,760 matching frames from the retest series (n = 13 pullbacks). Per frame analysis revealed that inter-study variability for mean lumen area and mean stent area was very low [resp. 0.01 ± 0.02 mm^2^ (0.19 %) and 0.01 ± 0.02 mm^2^ (0.11 %)]. Figure 3B shows Bland–Altman plots and regression analysis for these frames.

**Fig. 5:**
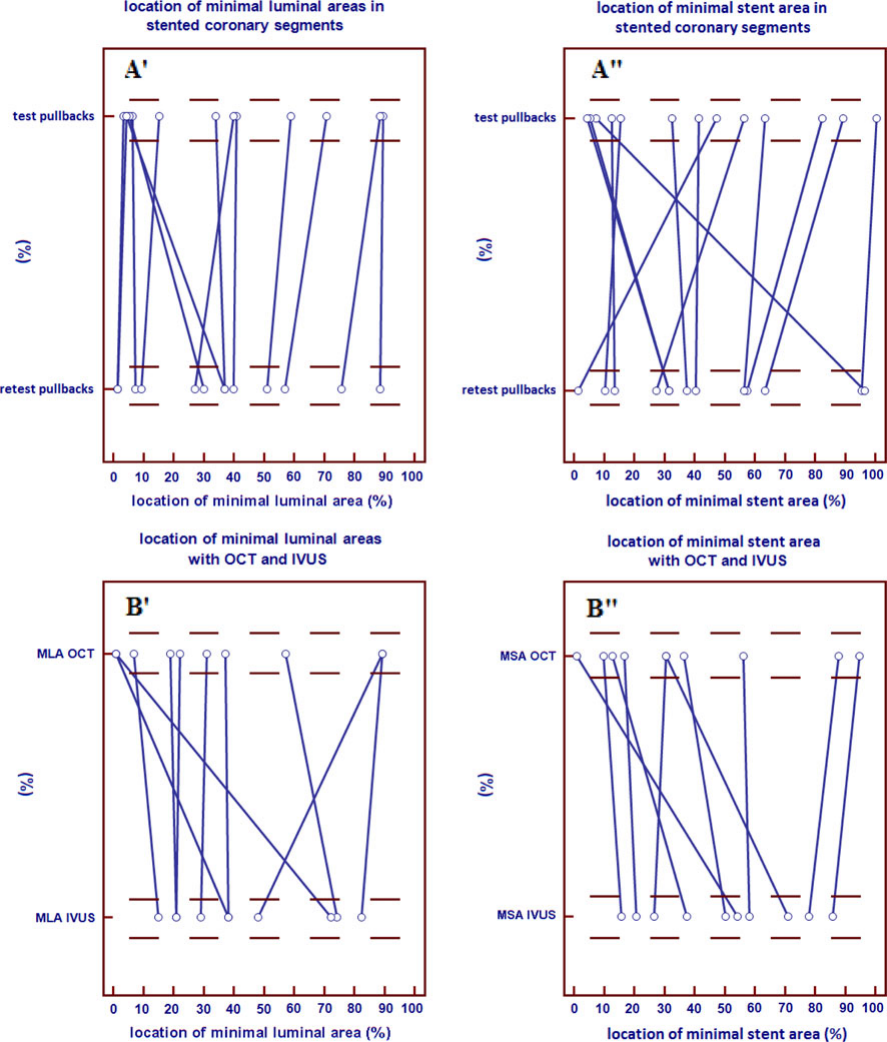
Comparison of the position of minimal luminal area (MLA) (**A′**, **B′**) and minimal stent are (MSA) (**A″**, **B″**) in stented coronary segments between two corresponding OCT pullbacks (**A′**, **A″**) and between OCT and IVUS (**B′**, **B″**). X-axis: length percentage of the total ROI/longitudinal position. Y-axis: corresponding pullbacks

Per stent analysis (mean test pullback length = 139 frames, mean retest pullback length = 139 frames) showed a very low inter-study variability for mean lumen area and mean stent area (0.06 ± 0.08, 0.07 ± 0.10 mm^2^, respectively). The inter-study variability for minimal luminal area and minimal stent area was 0.04 ± 0.09, 0.04 ± 0.10 mm^2^, respectively (Table 3). Relocation of the minimal luminal area and minimal stent area in stented coronary segments is shown in Fig. 5A. There were no significant relocations from MLA. There were five relocations of MLA with a difference >10 % in the longitudinal position between test and retest pullbacks, however the difference in MLA measurement was <10 % for these relocations. The biggest difference in MLA was 0.42 mm^2^ (9.27 mm^2^ in the test pullback and 9.69 mm^2^ in the retest pullback), this was not relocated. There were also no significant relocations from MSA. There were seven relocations of MSA with a difference >10 % in the longitudinal position between test and retest pullbacks, however the difference in MSA measurement was <10 % for these relocations. The biggest difference in MSA was 0.34 mm^2^ (6.75 mm^2^ in the test pullback and 6.41 mm^2^ in the retest pullback), this was not relocated.

**Table 3:** mean lumen area, mean stent area, minimal luminal area and minimal stent area in stented coronary segments in mm^2^

	Mean lumen area (mm^2^)	Mean stent area (mm^2^)	Minimal luminal area (mm^2^)	Minimal stent area (mm^2^)
OCT pullback 1	7.18 ± (1.37)	7.73 ± (1.09)	5.32 ± (1.30)	6.12 ± (0.85)
OCT pullback 2	7.12 ± (1.40)	7.66 ± (1.07)	5.36 ± (1.35)	6.08 ± (0.88)
Pullback 1 versus pullback 2				
Absolute difference	0.06 ± (0.08)	0.07 ± (0.10)	0.04 ± (0.09)	0.04 ± (0.10)
Relative difference	0.81 %	0.93 %	0.68 %	0.66 %
Linear regression				
Slope	0.98	1.01	0.96	0.97
Intercept	0.20	−0.03	0.16	0.24
R^2^	0.99	0.99	0.99	0.99
*P*	<0.001	<0.001	<0.001	<0.001

Malapposition (Fig. 2B) of stent struts was observed in 2 vessels; n = 1 LCx (n = 7 struts) and n = 1 RCA (n = 8 struts). In both stents, the malapposed struts were located at the proximal stent entrance. The mean malapposition area for the LCx stent was 0.28 mm^2^ and for the RCA stent 1.33 mm^2^. The inter-study variability for the malapposition area was 0.04 mm^2^ (14.2 %) for the LCx stent and 0.07 mm^2^ (5.2 %) for the RCA stent.

Tissue protrusion (Fig. 2C) was visible in n = 10 stents. The mean tissue protrusion area was 0.09 ± (0.04) mm^2^ in the test pullback series and 0.09 ± (0.04) mm^2^ in the retest pullback series, respectively. The inter-study variability for tissue protrusion was also very low [0.00 ± 0.02 mm^2^ (1.0 %)].

### Comparison to IVUS

**Fig. 6:**
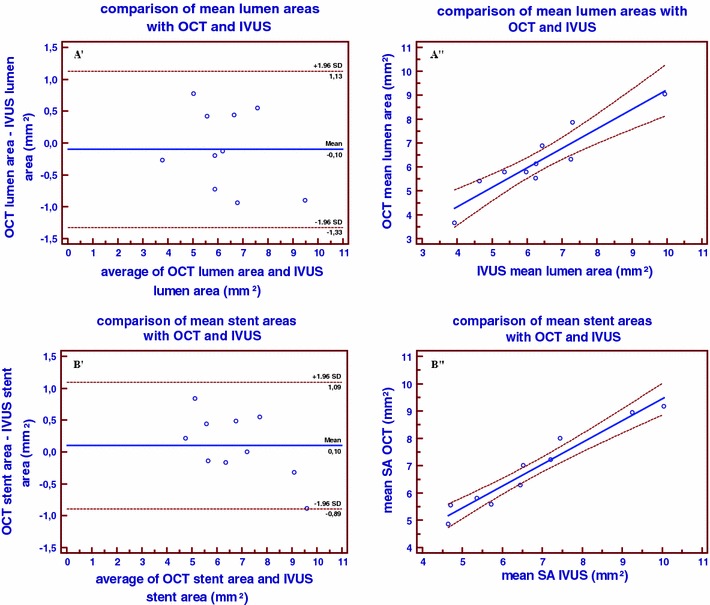
Bland Altman plots showing the differences in mean lumen areas (**A′**) and mean stent areas (**B′**) between OCT and IVUS in stented coronary arteries. Regression analyses lines showing correlation of mean lumen areas (**A″**) and mean stent areas (**B″**) between OCT and IVUS in stented coronary arteries

Comparison to IVUS morphometry revealed no significant differences between the invasive imaging methods. The differences between both methods were very low for mean lumen area, mean stent area, minimal luminal area and minimal stent area (0.10 ± 0.45, 0.10 ± 0.36, 0.26 ± 0.54, 0.05 ± 0.47 mm^2^, respectively) (Table 4). Bland–Altman plots and linear regression analysis for mean lumen area and mean stent area in stented coronary segments (n = 10) are shown in Fig. 6. Relocation of the minimal luminal area and minimal stent area in stented coronary segments is shown in Fig. 5B. There were no significant relocations of MLA. There were four relocations of MLA with a difference >10 % in the longitudinal position between OCT and IVUS pullbacks, however the difference in MLA measurement was <10 % for these relocations. The biggest difference in MLA was 1.52 mm^2^ (2.22 mm^2^ in the OCT pullback and 3.74 mm^2^ in the IVUS pullback), this was not relocated. There were also no significant relocations from MSA. There were four relocations of MSA with a difference >10 % in the longitudinal position between OCT and IVUS pullbacks, however the difference in MSA measurement was <10 % for these relocations. The biggest difference in minimal stent area was 1.69 mm^2^ (6.53 mm^2^ in the OCT pullback and 8.22 mm^2^ in the IVUS pullback), this was not relocated.

**Table 4:** OCT comparison to IVUS (per segment analysis)

	Mean lumen area (mm^2^)	Mean stent area (mm^2^)	Minimal lumen area (mm^2^)	Minimal stent area (mm^2^)
OCT pullback	6.24 ± (1.04)	6.84 ± (1.06)	4.59 ± (1.05)	5.31 ± (0.78)
IVUS pullback	6.34 ± (1.18)	6.74 ± (1.30)	4.84 ± (1.05)	5.35 ± (1.04)
Pullback 1 versus pullback 2				
Absolute difference	0.10 ± (0.45)	0.10 ± (0.36)	0.26 ± (0.54)	0.05 ± (0.47)
Relative difference	1.6 %	1.5 %	5.4 %	0.9 %
Linear regression				
Slope	0.82	0.80	0.86	0.68
Intercept	1.05	1.47	0.40	1.68
R^2^	0.86	0.94	0.75	0.82
*P*	<0.001	<0.001	=0.001	<0.001

IVUS was able to visualize strut malapposition in one stent located in the LCx, that was also observed by OCT. Mean malapposition area was 0.26 mm^2^ by both IVUS and OCT.

Tissue protrusion was visible in n = 7 stents by both IVUS and OCT. The mean protrusion area by IVUS was 0.09 ± 0.06 mm^2^ and by OCT was 0.11 ± 0.04 mm^2^. The difference between the mean protrusion area’s for OCT and IVUS was 0.02 ± 0.04 mm^2^ (19.7 %).

## Discussion

Our main findings in this study are: OCT shows very low inter-study variability in vivo and OCT shows no significant difference in quantitative lumen measurement as compared to IVUS. Because the FD-OCT technique is relatively new, little was known on the inter-study variability of this technique. Our study had two objectives: (a) to determine the inter-study variability of the intracoronary FD-OCT imaging system, and (b) to compare pullback images acquired by FD-OCT to the reference intravascular imaging method of IVUS within the same coronary segments.

OCT has rapidly been accepted as an alternative intracoronary imaging tool for the interventional cardiologist, due to more detailed information on atherosclerotic plaque pathology as well as on stent apposition and other repeatedly observed conditions after stent implementation such as dissection, tissue prolapse (in drug eluting stents), restenosis, fracture and thrombosis [2, 5, 7, 9, 19–23]. Given these advantages, OCT could be considered for application in studies evaluating new therapeutic treatments [24, 25].

### Interstudy variability

We found that the inter-study variability for lumen and stent area measurements with OCT using computer-assisted contour analysis is very low. The observed variability in our study is in the order of magnitude reported in previous studies for inter-observer and intra-observer reproducibility [8–11]. Our observations suggest that FD-OCT is a reliable imaging tool for the assessment of coronary artery disease, especially for longitudinal studies with repeated OCT examinations.

We were interested to understand to what extent relocation of the MLA is occurring, This question is of clinical interest especially in stented segments as the MLA as observed by invasive imaging might drive the decision for postdilatation, the balloon selection (in terms of diameter and lengths) and balloon position within the stent. In the native coronary series, there was no significant relocation observed, indicating that MLA by OCT is a robust parameter. The ROI length measured in number of frames, had a mean difference of 1 frame, which is 0.7 % of the mean pullback length. The mean longitudinal pullback speed relative to the coronary artery may be influenced, in principle, by cardiac motion. The finding of a small variation in ROI length indicates that it is sufficiently constant to perform reliable measurements in the vessel direction, which is a relevant observation in relation to stent sizing during PCI.

In our stented segments, no significant relocation was observed. Relocations with a difference in MLA or MSA of <10 % were observed, this can most likely be explained by a rather uniform stent expansion over the entire ROI. The incidence of relocations was increased compared to native vessels; however the absolute differences in lumen dimensions were very small due to the uniformity of stent expansion. Thus, they are unlikely to alter therapeutic decisions.

### Comparison to IVUS

Comparison of lumen and stent area morphometry for FD-OCT and IVUS did not demonstrate significant differences. However, OCT was able to visualize strut malapposition and tissue protrusion with higher sensitivity than IVUS. While Fourier domain OCT offers high resolution imaging at a high acquisition speed with a high contrast between the lumen and the arterial structures allowing OCT to visualize strut malapposition and tissue pretrusion better than IVUS [2, 26], it is hampered by its limited penetration depth. This often precludes the measurement of EEM and plaque area by OCT, especially in the presence of advanced atherosclerosis, which, however, is a strength of IVUS. There is controversy regarding differences in morphometry between both imaging techniques. A number of researchers reported no significant differences between both imaging techniques, while others reported a bias for IVUS with an overestimation of lumen and stent dimensions as compared to OCT. Kawamori et al. [26] reported no difference in lumen and minimal stent area for OCT compared to IVUS in patients. Kawasi et al. [27] reported no difference for lumen area and volume for OCT and IVUS in porcine coronaries. In contrast, others observed a tendency for smaller lumen areas when measured by OCT as compared to IVUS [3, 9, 28, 29].

The reason for these inconclusive findings is poorly understood. In most clinical studies, the sample size is rather small. Statistical significance should therefore be interpreted with caution. Morphometry by both technologies can be influenced by a number of variables including blood flow velocity, blood/flush media temperature and eccentric catheter placement [30]. We used X-ray contrast to clear artery form blood during OCT imaging and care was taken to maintain a temperature of 37 °C, while IVUS was performed under continuous blood flow. Flush delivery at constant flow rate could influence vasotonus, but the effect on scaffolded arteries is unclear.

Other possible explanations include differences in the delineation of the endoluminal border. OCT with its high resolution and the high contrast between the lumen and the vessel wall allows for clear visualization of the lumen, strut malapposition and tissue protrusion, while IVUS analysis can be hampered by the difficulties in differentiating the lumen border due to blood speckle or the presence of artifacts [28, 31]. Another reason for discrepancies could lay in differences in the analysis approaches, employed algorithms and the use of gating methods or not. Our findings in patients are in line with an in vitro study, Satoko et al. [45], who compared FD-OCT and IVUS in a coronary phantom model. They observed that FD-OCT was more accurate than IVUS in the phantom and showed better correlation with actual dimensions by OCT than IVUS.

### Limitations

The main limitation of our in vivo study was the small sample size, although more than 5,000 frames were included for analyses. As it was a clinical study, the guiding catheter was inserted only once, namely in the beginning of the procedure, we did not remove and re-insert the guiding catheter for the re-test series. Furthermore, we acknowledge the potential role of cofounders, including changes in blood pressure, heart rate, vascular tonus and intravascular pressure that might affect the inter-study variability. However, nitroglycerine was given to all patients before every pullback and no major changes in heart rate or blood pressure were observed.

A limitation of the study is the lack of inter-observer analysis and the fact that this is a single-centre study, which implies potential biases. However, we recently reported the observer-related variability of quantitative Fourier-domain OCT measurements in vivo. The intra-observer variance and coefficient of variation for lumen area on frame level was 0.0016 mm^2^ and 0.0052 respectively. The inter-observer variance and coefficient of variation for lumen area was 0.0003 mm^2^ and 0.0024 respectively [9].

Likewise, OCT demonstrated consistently low observer variability in various patient, lesion and experimental subsets. Generally, the reported inter- and intra- observer variability is very low, which might be explained by the high contrast in the OCT images, allowing to recognizes arterial structures easily.

Gonzalo et al. [10] examined the reproducibility of quantitative stent analysis. The relative difference for lumen area, stent area, tissue coverage area, tissue coverage thickness and strut coverage was around 1 % for the inter- and intra- observer reproducibility.

Tanimoto et al. [15] examined the observer-related variability of quantitative time-domain OCT measurements in both, in vitro and in vivo data. In vitro, the absolute and relative difference between lumen area measurements derived from two observers was 0.02 ± 0.10 mm^2^ and 0.3 ± 0.5 %, respectively with excellent correlation confirmed by linear regression analysis (R^2^ = 0.99; *P* < 0.001). In vivo, the absolute and relative difference between measurements were 0.11 ± 0.33 mm^2^ (1.57 ± 0.05 %) for lumen area (R^2^ = 0.98; *P* < 0.001), 0.17 ± 0.68 mm^2^ (1.44 ± 0.08 %) for stent area (R^2^ = 0.94; *P* < 0.001), and 0.26 ± 0.72 mm^2^ (14.08 ± 0.37 %) for neointimal area (R^2^ = 0.78; *P* < 0.001).

Gonzalo et al. analysed the inter- and intra- observer reproducibility for the diagnosis of qualitative features in coronary stents and plaque components. Kappa coefficients for strut malapposition were 0.83 and 0.83; for edge dissection 0.77 and 1.0, for tissue prolapse 0.78 and 1.0 and for intrastent dissection 1.0 and 1.0 for inter- and intra- observer, respectively. Plaque classification into main tissue components showed inter- observer agreement in the classification of 53 out of 60 plaques (k = 0.82; *P* < 0.001). The intra- observer variability showed agreement in the classification of 58 out of 60 plaques k = 0.95; *P* < 0.001) [8].

Okamura et al. [11] examined the inter software variability by comparing mean LA, mean SA, MLA based on corresponding cross sections. The absolute and relative differences between software packages were low, e.g. for lumen area 0.12 ± 0.10 mm^2^ and 1.98 ± 1.76 % (software 1 vs. software 2); 0.09 ± 0.10 mm^2^ and 1.43 ± 1.59 % (software 1 vs. software 3). Linear regression analysis confirmed these observations and showed a good correlation between measurements (R^2^ = 0.98–1.00).

Further, the z-offset was not automatically corrected but required manual calibration for every pullback. In the future algorithms for continuous, automated z-offset correction might reduce this source of variability.

We used two different IVUS systems for comparison with OCT. In the past, we reported a slight, systematic difference in lumen area measurements for phased array system as compared to a mechanical transducer system [32]. However, it warrants emphasis that the observed differences are comparable to those previously shown on intra and inter-observer variability for IVUS measurements [33]. Further, it remains unclear whether such variability is caused by an overestimation of measurements with the phased-array system, or by an underestimation by the mechanical system. Given this background, we assumed that potential difference in the used IVUS systems are within the order of magnitude of the measurement variability for IVUS and should, thus not significantly impact the comparison to OCT. Stratification of our data for the used IVUS system did not reveal a trend towards better or worse agreement with OCT in our series.

We cannot completely exclude a potential change in vascular tonus during intracoronary imaging, with potential lumen narrowing if the imaging procedure induces spasm or potential distension of the lumen if the imaging increases intra-arterial pressure.

In order to avoid the first, we apply NTG ic. before every imaging run in a standardized fashion. In order to avoid the latter, we apply a standard flush protocol injecting X-ray contrast medium (Iodixanol 370) at 37 °C with a flow rate of 3 ml/s. Care is taken that the guide catheter is in a co-axial position and that no wedging is observed. Such flush protocol increases the intra-arterial pressure by typically 10 mmHg, which is not expected to induce significant lumen changes [29].

## Conclusion

FD-OCT shows excellent reproducibility and very low inter-study variability in both, native and stented coronary segments. No significant differences in lumen morphometry were observed between FD-OCT and IVUS.

## References

[1] Regar E, Schaar JA, Mont E, Virmani R, Serruys PW (2003). Optical coherence tomography. Cardiovasc Radiat Med.

[2] Bouma BE, Tearney GJ, Yabushita H, Shishkov M, Kauffman CR, DeJoseph Gauthier D (2003). Evaluation of intracoronary stenting by intravascular optical coherence tomography. Heart.

[3] Yamaguchi T, Terashima M, Akasaka T, Hayashi T, Mizuno K, Muramatsu T (2008). Safety and feasibility of an intravascular optical coherence tomography image wire system in the clinical setting. Am J Cardiol.

[4] Asawa K, Kataoka T, Kobayashi Y, Hasegawa T, Nishioka H, Yamashita H (2006). Method analysis for optimal continuous imaging using intravascular optical coherence tomography. J Cardiol.

[5] Tearney G, Waxman S, Shishkov M, Vakoc BJ, Suter MJ, Freilich MI (2008). Three-dimensional coronary artery microscopy by intracoronary optical frequency domain imaging. J Am Coll Cardiol Img.

[6] Tahara S, Bezerra HG, Baibars M (2011). In vitro validation of new fourier-domain optical coherence tomography. Eurointervention.

[7] Prati F, Zimarino M, Stabile E, Pizzicannella G, Fouad T, Rabozzi R (2008). Does optical coherence tomography identify arterial healing after stenting? An in comparison with histology, in a rabbit carotid model. Heart.

[8] Gonzalo N, Tearney GJ, Serrruys PW, van Soest G, Okamura T, Garcia–Garcia HM, van Geuns RJ, van der Ent M, Ligthart J, Bouma BE, Regar E (2010). Second generation optical coherence tomography in clinical practice. High-speed data acquisition is highly reproducible in patients undergoing percutaneous coronary intervention. Rev Esp Cardiol.

[9] Okamura T, Onuma Y, Garcia-Garcia HM, van Geuns RJ, Wykrzykowska JJ, Schultz C, van der Giessen WJ, Ligthart J, Regar E, Serruys PW (2011). First-in-man evaluation of intravascular optical frequency domain imaging (OFDI) of Terumo: a comparison with intravascular ultrasound and quantitative coronary angiography. EuroIntervention.

[10] Gonzalo N, Garcia Garcia H, Serruys PW, Commissaris K, Gobbens P, Costa MA, Regar E (2009). Reproducibility of quantitative strut stent analysis with optical coherence tomography. EuroIntervention.

[11] Okamura T, Gonzalo N, Gutierres Chico JL, Serruys PW, Bruining N, de Winter S, Dijkstra J, Commissaris KH, van Geuns RJ, van Soest G, Ligthart J, Regar E (2010). Reproducibility of coronary Fourier domain optical coherence tomography: quantitative analysis of in- vivo stented coronary arteries using three different software packages. EuroIntervention.

[12] Yun SH, Tearney GJ, Vakoc BJ, Shishkov M, Oh WY, Desjardins AE, Suter MJ, Chan RC, Evans JA, Jang IK, Nishioka NS, de Boer JF, Bouma BE (2007). Comprehensive volumetric optical microscopy in vivo. Nat Med.

[13] Vakoc B, Yun S, de Boer J, Tearney G, Bouma B (2005). Phase-resolved optical frequency domain imaging. Opt Express.

[14] Sihan K, Botha C, Post F, de Winter S, Gonzalo N, Regar E, Serruys PW, Hamers R, Bruining N (2009). Fully Automatic three-dimensional (3D) quantitative analysis of intracoronary optical coherence tomography: method and validation. Catheter Cardiovasc Interv.

[15] Tanimoto S, Rodriguez-Granillo G, Barlis P, de Winter S, Bruining N, Hamers R, Knappen M, Verheye S, Serruys PW, Regar E (2008). A novel approach for quantitative analysis of intracoronary optical coherence tomography: high inter-observer agreement with computer-assisted contour detection. Catheter Cardiovasc Interv.

[16] Regar E, Werner F, Siebert U, Rieber J, Thesen K, Mudra H, Klauss V (2000). Reproducibility of neointima quantification with motorized intravascular ultrasound pullback in stented coronary arteries. Am Heart J.

[17] De Winter SA, Hamers R, Degertekin M, Tanabe K, Lemos PA, Serruys PW, Roelandt JR, Bruining N (2004). Retrospective image-based gating of intracoronary ultrasound images for improved quantitative analysis: the intelligate method. Catheter Cardiovasc Interv.

[18] Bland JM, Altman DG (1986). Statistical methods for assessing agreement between two methods of clinical measurement. Lancet.

[19] Barlis P, Sianos G, Ferrante G, Del Furia F, D’Souza S, Di Mario C (2009) The use of intra-coronary optical coherence tomography for the assessment of sirolimus-eluting stent fracture. Int J Cardiol 136(1):e16–20 10.1016/j.ijcard.2008.04.076 18723234

[20] Yabushita H, Bouma BE, Houser SL, Aretz HT, Jang IK, Schlendorf KH (2002). Characterization of human atherosclerosis by optical coherence tomography. Circulation.

[21] Tearney GJ, Yabushita H, Houser SL, Aretz HT, Jang IK, Schlendorf KH (2003). Quantification of macrophage content in atherosclerotic plaques by optical coherence tomography. Circulation.

[22] Gonzalo N, Serruys PW, Okamura T, van Beusekom HM, Garcia–Garcia HM, van Soest G (2009). Optical coherence tomography patterns of stent restenosis. Am Heart J.

[23] Gonzalo N, Garcia–Garcia HM, Regar E, Barlis P, Wentzel J, Onuma Y (2009). In vivo assessment of high-risk coronary plaques at bifurcations with combined intravascular ultrasound and optical coherence tomography. JACC Cardiovasc Imaging.

[24] Guagliumi G, Sirbu V (2008). Optical coherence tomography: High resolution intravascular imaging to evaluate vascular healing after coronary stenting. Catheter Cardiovasc Interv.

[25] Ormiston JA, Serruys PW, Regar E, Dudek D, Thuesen L, Webster MW, Onuma Y, Garcia–Garcia HM, McGreevy R, Veldhof S (2008). A bioabsorbable everolimus-eluting coronary stent system for patients with single de novo coronary artery lesions (ABSORB): a prospective open-label trial. Lancet.

[26] Kawamori H, Shite J, Shinke T, Otake H, Sawada T, Kato H (2010). The ability of optical coherence tomography to monitor percutaneous coronary intervention: detailed comparison with intravascular ultrasound. J Invasive Cardiol.

[27] Kawase Y, Hoshino K, Yoneyama R, McGregor J, Hajjar RJ, Jang IK (2005). In vivo volumetric analysis of coronary stent using optical coherence tomography with a novel balloon occlusion-flushing catheter: a comparison with intravascular ultrasound. Ultrasound Med Biol.

[28] Suzuki Y, Ikeno F, Koizumi T, Tio F, Yeung AC, Yock PG (2008). In vivo comparison between optical coherence tomography and intravascular ultrasound for detecting small degrees of in-stent neointima after stent implantation. J Am Coll Cardiol Interv.

[29] Gonzalo N, Serruys PW, Garcia–Garcia HM, van Soest G, Okamura T, Ligthart J, Knaapen M, Verheye S, Bruining N, Regar E (2009). Quantitative ex vivo and in vivo comparison of lumen dimensions measured by optical coherence tomography (OCT) and intravascular ultrasound (IVUS) in human coronary arteries. Rev Esp Cardiol.

[30] Chae JS, Brisken AF, Maurer G, Siegel RJ (1992). Geometric ac- curacy of intravascular ultrasound imaging. J Am Soc Echocardiogr.

[31] Gonzalo N, Serruys PW, Okamura T, Shen ZJ, Onuma Y, Garcia–Garcia HM (2009). Optical coherence tomography assessment of the acute effects of stent implantation on the vessel wall: a systematic quantitative approach. Heart.

[32] Rodriguez-Granillo GA, McFadden EP, Aoki J, van Mieghem CA, Regar E, Bruining N, Serruys PW (2006). In vivo variability in quantitative coronary ultrasound and tissue characterization measurements with mechanical and phased-array catheters. Int J Cardiovasc Imaging.

[33] Hausmann D, Lundkvist A-J, Friedrich GJ, Mullen WL, Fitzgerald PJ, Yock PG (1994). Intracoronary ultrasound imaging: Intraobserver and interobserver variability of morphometric measurements. Am Heart J.

[34] Barlis P, Regar E, Serruys PW, Dimopoulos K, van der Giessen WJ, van Geusn RJM, Ferrante G, Wandel S, Windecker S, van Es GA, Eerdmans P, Jüni P, di Mario C (2010). An optical coherence tomography study of a biodegradable versus durable polymer-coated limus-eluting stent: a LEADERS trial sub-study. Eur Heart J.

